# Sex differences in human adipose tissues – the biology of pear shape

**DOI:** 10.1186/2042-6410-3-13

**Published:** 2012-05-31

**Authors:** Kalypso Karastergiou, Steven R Smith, Andrew S Greenberg, Susan K Fried

**Affiliations:** 1 Department of Medicine, Section of Endocrinology, Diabetes & Nutrition, Boston University School of Medicine, Boston, MA, USA; 2 Diabetes and Obesity Research Center, Sanford/Burnham Medical Research Institute at Lake Nona, Orlando, FL and Translational Research Institute for Metabolism and Diabetes, Florida Hospital, Sanford/Burnham Medical Research Institute at Lake Nona, Orlando, FL, USA; 3 Human Nutrition Research Center on Aging, Tufts University, Boston, MA, USA; 4 Division of Endocrinology, Diabetes, and Nutrition, Department of Medicine, Boston University, School of Medicine, 650 Albany St, EBRC-810, Boston, MA, 02118, USA

**Keywords:** Adipocyte, Fat distribution, Lipolysis, Fatty acid uptake

## Abstract

Women have more body fat than men, but in contrast to the deleterious metabolic consequences of the central obesity typical of men, the pear-shaped body fat distribution of many women is associated with lower cardiometabolic risk. To understand the mechanisms regulating adiposity and adipose tissue distribution in men and women, significant research attention has focused on comparing adipocyte morphological and metabolic properties, as well as the capacity of preadipocytes derived from different depots for proliferation and differentiation. Available evidence points to possible intrinsic, cell autonomous differences in preadipocytes and adipocytes, as well as modulatory roles for sex steroids, the microenvironment within each adipose tissue, and developmental factors. Gluteal-femoral adipose tissues of women may simply provide a safe lipid reservoir for excess energy, or they may directly regulate systemic metabolism via release of metabolic products or adipokines. We provide a brief overview of the relationship of fat distribution to metabolic health in men and women, and then focus on mechanisms underlying sex differences in adipose tissue biology.

## Review

Women, compared to men, have higher percent body fat and deposit it in a different pattern, with relatively more adipose tissue in the hips and thighs. This ‘female’ fat distribution, independent of total body fat, confers protection against metabolic diseases, such as type 2 diabetes and atherosclerosis [1]. Although sex differences in fat distribution and correlations to metabolic health are well established in the clinical and epidemiological literatures [1,2], the biological underpinnings of these associations remain poorly understood. Microarray analyses show that adipose mass and depot differences in adipose tissue gene expression in mice are regulated by sexually dimorphic gene networks. Inflammatory and developmental genes, some of which are modulated by sex steroid hormones, are prominent among depot- and sex-specific genes [3-5]. Furthermore, and especially important for understanding the pathogenesis of obesity and its metabolic complications, interactions of sex differences in gene expression with environmental variables such as diet composition and exercise/activity on fatness and fat distribution remain largely unexplored. Because excellent reviews of sex differences in the regulation of food intake and body weight have been recently published [6,7], in this review, we focus on physiologic and genetic determinants of sex differences in fat distribution.

### The adipose organ of humans

Cinti convincingly argues that body fat is stored in the adipose organ which consists of definable fat depots [8]. Subcutaneous white adipose tissue (SAT) depots, just under the skin, store ~80-90% of total body fat, mainly in the abdominal (around the waist), subscapular (on the upper back), gluteal and femoral (thigh) areas. These subcutaneous adipose tissues have distinct morphological and metabolic profiles and exhibit sex-specific differences in size and function that we will review in detail. Intra-abdominal depots include visceral adipose tissues (VAT, omental and mesenteric), which are associated with digestive organs. VATs drain their blood into the portal vein and account for 6-20% of total body fat, with higher values in males than females [9-12]. Adipose tissues in the retroperitoneal compartment (~7% of total [13]) do not drain into the portal vein and are therefore not considered ‘visceral’.

It has been recently recognized that humans, even in adulthood, possess islands of brown adipose tissue, mainly in the supraclavicular/dorsal cervical area [14,15]. Whereas the function of white adipose tissues is to store excess energy, the function of brown fat is to produce heat. Indeed brown fat mass and activity are induced by cold stress [16]. The exact role of this specialized tissue in the regulation of energy balance in humans remains to be determined ([16], and reviewed in [17]). Of interest with regard to sex differences in metabolism, limited data indicate that women tend to have higher quantities of brown fat, but the significance of this observation is not clear, as women do not have higher energy expenditure (adjusted for lean mass) [14,15,18]. The regulation of brown adipose tissue mass and function in determining susceptibility to obesity in men and women is currently an active and important area of investigation, but little is known. This review will focus on sex differences in human white adipose tissues.

#### Determinants of sex differences in body fat and fat distribution

For the same body mass index (BMI), women typically present with ~10% higher body fat compared to men [19,20]. Aging increases adiposity in both sexes, but again, women are characterized by higher percent body fat throughout the entire life span [21]. Sex x race interactions are also evident: in contrast to Caucasians, African American women and men appear to have comparable fat content at higher BMI levels [22].

At comparable levels of total adiposity (estimated either from BMI or by imaging techniques), women have more SAT both in the abdominal [22-25] and in the gluteofemoral area [26,27]. Moreover, this is due to preferential increase of superficial and not deep SAT in women; it is the size of the deep compartment that is inversely associated with fasting insulin levels [28]. At the same time women are characterized by lower intra-abdominal/visceral fat mass [23-25]. However, the difference in visceral fat mass is diminished and not consistently seen in older age groups [22,29]. Women of Asian origin, either Chinese or South Asian, have higher VAT content than Caucasian women (BMI-adjusted), but lower than men of the same ethnicity [30]. Only black women have VAT comparable to and at the same time abdominal SAT larger than black men [24,27].

### Fat distribution is modulated by sex steroids

Striking sex differences arise during puberty: the increase in body weight in boys is primarily due to increases in lean mass whereas in girls due to increases in fat mass; typical android and gynoid fat distributions also appear for the first time during this time period [31-33]. Menopause is followed by redistribution of adipose tissue towards a more central/android phenotype [34,35]. Importantly, it is visceral adiposity that rises in women during the peri-menopausal transition, presumably due to the fall in estrogen levels [36,37]. As testosterone declines with age, visceral adiposity also increases in men [38,39]. The hyperandrogenism in women with polycystic ovary syndrome is frequently, but not consistently, associated with increased total and abdominal adiposity [40-42]. Finally, studies of transgendered men and women treated with sex steroids show clear shifts in fat distribution [43]. Very little is understood about the cellular and molecular mechanisms by which sex steroids modulate the growth and metabolism and hence the size of specific fat depots in humans (as reviewed below).

### Genetic determinants of total adiposity and distribution in women and men

Twin studies demonstrate that genetic factors account for up to 70% of the BMI variance [44], and that this effect is influenced by sex [45]. A few rare genetic syndromes have differential effects on total adiposity in males and females and may provide clues to understanding sex differences in adiposity [46-49]. A number of polymorphisms in the estrogen receptor α gene are associated with total adiposity and fat distribution and in some cases this relationship is restricted to females [50-52]. Results of recent genome-wide association studies (GWAS) have identified genetic determinants of common polygenic obesity that interact with environmental variables in complex ways, but so far explain only a small percentage of the inter-individual variation in BMI [53].

GWAS and meta-analyses of GWAS have also identified novel loci associated with central or peripheral fat distribution, some of which are sex-specific (summarized in Table 1) [54,55]. For five of these loci (near or in *RSPO3, TBX15, ITPR2, WARS2* and *STAB1)*, differential mRNA expression is also noted between abdominal and gluteal tissue [54]. Although the functional correlates of these SNPs are yet to be identified, there are a number of intriguing candidates. For example, VEGF plays an important role in the vascularization of the expanding adipose tissue in development or obesity [56]; GRB-14 inhibits insulin action [57,58]; TFAP2B affects adipokine secretion and adipocyte insulin sensitivity [59,60] and TBX15 differentiation and lipid accumulation [61]. Both KREMEN1 and RSPO3 interact with the Wnt signaling pathway, which in turn plays a fundamental role in adipocyte differentiation [62,63].

**Table 1:** Single nucleotide polymorphisms (SNPs) associated with fat distribution in genome-wide association studies

**SNP**	**Nearby gene**	**Associated with**	**Significant in men**	**Significant in women**	**Significant sex difference**
rs9491696	*RSPO3* (R-spondin 3)	WHR	Yes	Yes	Yes
rs6905288	*VEGFA* (vascular endothelial growth factor A)	WHR	Yes	Yes	Yes
rs2605100	*LYPLAL1* (lysophospholipase-like protein 1)	WHR	No	Yes	Yes
rs4846567		WHR	No	Yes	Yes
rs718314	*ITPR2* (inositol 1,4,5-triphosphate receptor 2) – *SSPN* (sarcospan)	WHR	Yes	Yes	Yes
rs1443512	*HOXC13* (homeobox C13)	WHR	Yes	Yes	Yes
rs4823006	*ZNRF3* (zinc and ring finger 3) – *KREMEN1* (kringle containing transmembrane protein 1)	WHR	Yes	Yes	Yes
rs10195252	*GRB14* (growth factor receptor-bound protein 14)	WHR	Borderline	Yes	Yes
rs6795735	*ADAMTS9* (ADAM metallopeptidase with thrombospondin type 1 motif, 9)	WHR	Borderline	Yes	Yes
rs984222	*TBX15* (T-box 15) – *WARS2* (tryptophanyl tRNA synthetase 2, mitochondrial)	WHR	Yes	Yes	No
rs1055144	*NFE2L3* [nuclear factor (erythroid-derived 2)-like 3]	WHR	Yes	Yes	No
rs1011731	*DNM3* (dynamin 3) – *PIGC* (phosphatidylinositol glycan anchor biosynthesis, class C)	WHR	Yes	Yes	No
rs1294421	*LY86* (lymphocyte antigen 86)	WHR	Yes	Yes	No
rs6784615	*NISCH* (nischarin) – *STAB1* (stabilin 1)	WHR	Yes	Yes	No
rs6861681	*CPEB4* (cytoplasmic polyadenylation element binding protein 4)	WHR	Yes	Yes	No
rs987237	*TFAP2B* (transcription factor activating enhancer-binding protein 2 beta)	WC	Yes	Yes	No
rs7826222	*MSRA* (methionine sulfoxide reductase A)	WC	Yes	Yes	No

*WHR* waist-to-hip ratio, *WC* waist circumference.

These loci explain in total only 1.34% of the variance in waist-to-hip ratio in women, and even less (0.46%) in men [54]. Undoubtedly, epigenetic regulation of gene expression by environmental and/or hormonal factors contributes significantly to individual variation and sex differences in fat distribution. Animal studies show that exposure to sex steroids early in life alters adipose tissue distribution and function in adulthood [64,65]. In humans, sex differences in epigenetic regulation have been reported in several tissues [66-69] and are feasible to test in adipose tissue [70]. Recent studies showing that acute exercise, overfeeding, and type 2 diabetes can modulate gene expression in skeletal muscle through epigenetic mechanisms [71-73] open the exciting scenario that lifestyle factors can interact with developmental programming to regulate adipose tissue mass and distribution.

### Metabolic significance of body fat distribution

#### Body fat distribution is linked to health in both men and women

Since the seminal work of Jean Vague, it has become clear that sex differences in adiposity and fat distribution are closely associated with whole body metabolism and long-term health [74,75]. Thus, although BMI is in itself a strong predictor of total mortality [76], this is far from a simple, universal relationship. Certain individuals retain metabolic health despite being morbidly obese [77], while others develop disease at paradoxically normal adiposity levels [78]. In both sexes, a peripheral body fat distribution clearly dissociates fat mass from risk for metabolic diseases [79,80].

#### Gluteal-femoral fat distribution is associated with lower metabolic risk

The clinical significance of body fat distribution is supported by multiple epidemiological studies that confirm the detrimental effect of central body and the protective effect of gluteal-femoral fat on diabetes [81,82], cardiovascular risk and eventually morbidity and mortality [1,76,83-89]. Early clinical studies based on anthropometric measurements also showed very clearly that the protective peripheral fat distribution phenotype (pear shape) is mainly seen in women [90]. However, ~40% of women between the age of 30–79 store fat predominantly in the abdominal area as evident by a waist-to-hip ratio >0.85 [91]. These so-called upper body obese women suffer from the same metabolic complications as men [92].

As recently reviewed [93], premenopausal women, compared to age-matched men, have better lipid profiles: higher high density lipoprotein (HDL)-cholesterol levels and lower low density lipoprotein (LDL)-cholesterol, very low density lipoprotein (VLDL)-cholesterol and total triglyceride levels. Importantly, this improved lipid profile is seen both in the fasting state and postprandially [94,95] and the sex differences cannot be attributed solely to preferential VAT accumulation in men [96]. Although fasting insulin concentrations are comparable between BMI-matched men and women [97], women show improved insulin sensitivity at the level of liver and skeletal muscle (reviewed in [98]). This apparent contradiction between higher total body fat and improved systemic metabolism in women intuitively leads to questions about sex differences in the biology and function of different adipose tissue depots.

#### Mechanisms linking gluteal-femoral fat to metabolic risk are unclear

The storage capacity of gluteal-femoral adipose tissues may play a role in determining the level of central adiposity. A recent randomized-controlled trial in nonobese women showed that removal of thigh fat by liposuction is followed by re-accumulation preferentially in the abdominal area, suggesting that effective peripheral fat storage may protect from the expansion of central fat depots [99]. This ‘redistribution’ of fat would be expected to have an impact on metabolic risk over the long term. However, it is also possible that gluteal-femoral adipose tissue plays an active role in metabolism. Studies in male mice show that transplantation of inguinal SAT as compared to epididymal (which has properties of visceral depots) inside the abdominal cavity leads to less body weight gain and better glucose tolerance [100,101]. On the other hand, other laboratories report that transplantation of epididymal fat into the abdominal cavity also improves glucose tolerance [102,103]. Whether SAT of female mice has more potent effects has yet to be reported, and we are currently undertaking these studies.

### Sex differences in adipose tissue metabolic function

**Figure 1:**
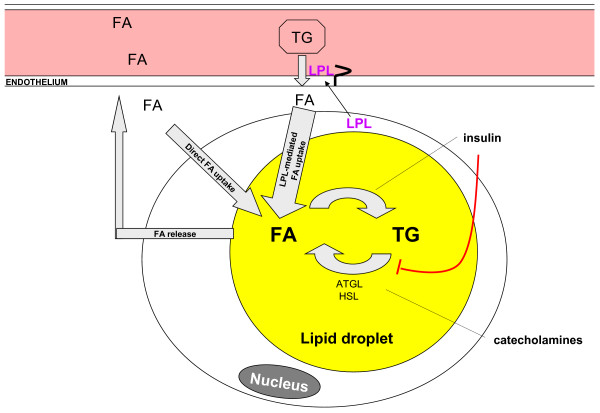
**Simplified overview of adipocyte metabolism.** After a meal, triglycerides (TG) packaged into chylomicrons are transported to the adipocytes. The enzyme lipoprotein lipase (LPL) made in the adipocyte is secreted to the capillary endothelium were it cleaves TG to fatty acids (FA) which are in turn taken up by the adipocyte and esterified to a backbone of alpha-glycerophosphate (which is mainly derived from glucose). Insulin stimulates this process, mainly by stimulating glucose uptake. Stored TG can be mobilized after hydrolysis by lipases (adipose tissue triglyceride lipase - ATGL and hormone sensitive lipase - HSL). The process of lipolysis is stimulated by catecholamines and inhibited by insulin. Gluteo-femoral adipocytes of women are more efficient in storing FA via the direct pathway and also show higher LPL activity. See text for details.

The major function of adipose tissue is the appropriate and highly regulated storage and release of energy (Figure 1). Free fatty acids (FFA), either circulating or derived from chylomicrons, VLDL-cholesterol and triglycerides (TG) by the action of lipoprotein lipase (LPL), are taken up by the adipocytes and stored as intracellular TG, three fatty acids esterified to a glycerol backbone. Stored TG can be mobilized as required between meals and in the starved state. Insulin and catecholamines act as the main regulatory signals of the fed and the fasted state respectively. The integrated regulation of TG storage and mobilization together with the endocrine function of adipose tissue are essential for whole body metabolism as reviewed before [104]. Depot differences in abdominal versus gluteofemoral fat have been reviewed in detail [1,93] so here we will emphasize the sex-specific characteristics.

#### Storage of energy in adipose tissue: subtle differences between sexes

It would be reasonable to expect that women are more effective in storing fat subcutaneously and men intra-abdominally, as that could explain preferential fat deposition. Indeed early *in vitro* studies suggested that subcutaneous adipocytes/adipose tissue from women show higher LPL activity [105], lipid synthesis [106] and insulin-stimulated glucose uptake [107,108] compared to men. *In vivo* studies however show that the answer is more complex.

Women store a higher percentage of meal-derived FA in SAT compared to men (38% vs 24%) [109,110], but this is a direct result of their greater SAT mass. When expressed as per gram of adipose tissue lipid, i.e. per unit of fat mass, FA uptake is comparable between sexes in all three SAT depots (abdominal, gluteal and thigh) [109]. On the other hand, the uptake of meal-derived fatty acids by VAT in men exceeds that in women, whether it is calculated as % of total meal disposal or as absolute values (μmol/min) [111]. Thus, differences in FFA storage after a meal are likely to contribute to VAT expansion in men, but less likely to contribute to SAT expansion in women.

Under certain conditions though, the female tendency to store fat in the peripheral subcutaneous depots becomes more apparent. In response to a hypercaloric, high-fat meal, storage of meal-derived triglyceride-fatty acids *per gram of adipose tissue lipid* is increased preferentially in the gluteo-femoral SAT of women compared to men [112], despite the fact that the adipocytes are larger, i.e. there are fewer adipocytes per gram. Additionally, meal fat storage is more efficient in peripheral vs. abdominal SAT of women with gluteal-femoral obesity; in upper body obese women or obese men there are no such regional differences [113]. These findings appear to be driven by depot differences in LPL activity [111], which is a rate-determining step in the uptake of circulating triglycerides. Further studies are needed to assess the mechanisms for these depot- and sex-specific phenomena, including assessment of regional differences in sex steroid action on LPL [114-116] in men and women of varying fat distribution.

Net fat storage in the adipose tissue after a meal requires inhibition of lipolysis, achieved by increased circulating insulin levels. This mechanism is less effective both in men and in upper body obese women [117,118] compared to women with peripheral fat distribution. Differences in insulin sensitivity (higher in females) are reproduced *in vitro* in isolated adipocytes of pre- [119], but not post-menopausal women [120] and can also be causally linked to the detrimental postprandial metabolic profile seen in men and upper body obese women.

Although it was once believed that LPL-mediated uptake of fatty acids from circulating TG-rich lipoproteins at the fed state is the major or sole mechanism for FA provision to the adipose tissue, the importance of direct uptake of circulating FFA at the postabsorptive state is now realized [121]. This pathway shows clear sexual dimorphism: at the whole body level, women deposit double the percentage of circulating FFA (8.2% versus 4.0%) in body fat. Furthermore, lean men are less efficient at depositing circulating FFA into femoral as compared to abdominal adipose tissue, while lean women show no depot difference [121]. In obese subjects direct FFA uptake is enhanced specifically in the femoral tissue of women [121]. The peripheral fat depots of women are also more effective in FFA storage during physical activity (walking) in comparison to both abdominal SAT of women or to any SAT depot of men [122]. Higher direct FFA storage was also noted in the omental adipose tissue of women (a visceral depot), which is opposite to what would be predicted from the lower mass of this depot in women. Therefore, the capacity for direct FFA deposition is likely not the major determinant of visceral adiposity [123].

**Figure 2:**
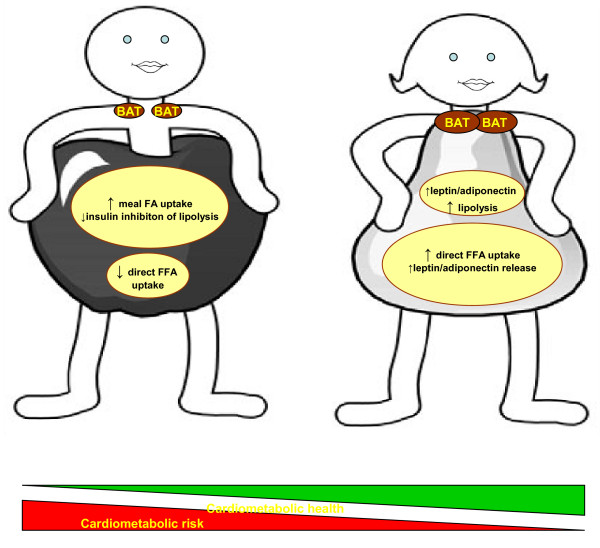
**Key sex differences in adipose tissue.** Compared to men, women are characterized by increased amounts of brown adipose tissue BAT and enlarged peripheral fat depots, whereas intra-abdominal fat depots are preferentially increased in men. Sex differences in the metabolic and endocrine function of these depots are associated with diminished disease risk in women. *FFA* free fatty acids.

To summarize, differences in fat deposition between sexes are likely to arise partly due to: a) the preferential postabsorptive direct FFA uptake by SAT in women and b) the enhanced postprandial meal-derived FFA uptake by VAT in men (Figure 2).

#### Release of energy from adipose tissue: female adipose tissue is more lipolytically active

To achieve normal fatty acid homeostasis, the FFA flux to or from adipose tissue needs to match whole body energy requirements. FA release in excess of the needs of other tissues leads to elevated circulating FFA levels or flux, which in turn contribute to insulin resistance, ectopic lipid accumulation and lipotoxicity. Thus, it comes as no surprise that total FFA flux correlates closely with whole body energy requirements (resting energy expenditure [124]). Given the higher fat levels in women, one could hypothesize that release of FFA (lipolysis) would be suppressed compared to men. On the contrary, lipolysis relative to resting energy expenditure is significantly higher (by about 40%) in women [124]. This is achieved without deleterious consequences partly because women are more dependent on fat oxidation than men in periods of high energy requirements like exercise, when men tend to utilize more carbohydrates [125]. Therefore, the increased lipolysis is a mechanism that matches well the preferential use of FFA in women.

On the other hand, lipolytic rates in women are higher than in men even under resting conditions when FA oxidation is comparable between sexes [125]. This is associated with ~15% higher levels of circulating FFA levels [126] but *not* with any detrimental effects on whole body metabolism [127,128]. It follows logically that for women to preserve their insulin sensitivity, they have to be more effective in alternative FFA disposal [124]. Indeed, recent studies demonstrated that women exhibit higher non-oxidative FFA disposal (i.e. esterification and storage as triglycerides) [129] and after an overnight fast, they preferentially partition FFA towards hepatic oxidation to ketone bodies, rather than incorporation into VLDL-TG [130]. FFA can also be shuttled back to adipose tissue through the direct FFA uptake pathway discussed above.

Sex differences in systemic lipolysis appear to arise at the level of the upper-body SAT which is, in both sexes, the main source of circulating FFA [117,131,132]. Norepinephrine-stimulated lipolysis in abdominal SAT in women exceeds that seen in men both *in vivo* and *ex vivo*[133,134]. Similarly, after exercise, the increase in circulating glycerol is augmented in women compared to men [125,135], as well as the glycerol release specifically by the abdominal SAT (no differences were seen between sexes in the gluteofemoral SAT) [135,136]. Prolonged fasting (up to 72 h) leads to exacerbated stimulation of lipolysis in women compared to men, despite comparable rises in catecholamine levels [137,138]. The opposite holds true for VAT, where *ex vivo* and *in vivo* stimulation of lipolysis is higher in men [134,139,140], but this has more impact on FA flux to the liver via the portal circulation and little impact on systemic FFA flux. Finally in men, e*x vivo* lipolysis is higher in intra-peritoneal (omental and mesenteric) than retro-peritoneal depots, while the opposite occurs in women, but the *in vivo* physiological significance of these observations is unclear [139].

To summarize: women, compared to men, show higher rates of mobilization of adipose tissue TG stores, possibly because they are more dependent on FFA as an energy source under conditions of high energy demands like exercise. At the same time they are more efficient in handling FFA and thus retain their insulin sensitivity. Depot differences in lipolysis however cannot explain the peripheral deposition of fat in women compared to men (Figure 2).

### Sex differences in the endocrine function of adipose tissue

Apart from regulating fuel homeostasis, adipose tissue releases a multitude of secretory products, collectively called adipokines. The regulation of adipokine release and their individual roles have been reviewed extensively ([141,142] among others). The two major adipokines are leptin, a metabolic regulator and feedback signal of body fat to regulate appetite, and adiponectin, an insulin-sensitizing and anti-inflammatory hormone. Multiple studies have established that women have higher circulating leptin levels compared to men, even after adjusting for differences in BMI and body fat content [143,144] and this finding is replicated in *ex vivo* adipose tissue cultures [145]. Interestingly, although sex differences in leptin are augmented during puberty, they are also apparent in children and even in neonates [146-148]. Leptin levels do not decline with menopause suggesting again that the higher leptin relative to body fat in women is not simply due to sex steroids (although postmenopausal women have slightly lower leptin per kg of fat compared to premenopausal women, they still have higher levels in comparison to men) [149,150]. Higher circulating adiponectin concentrations are also seen in women despite their higher adiposity, which is associated in both sexes with lower adiponectin levels [151-153]. These sex differences in adipokine production are of dual significance. They suggest inherent differences in adipocyte function between sexes or differential regulation by hormones, e.g. suppressive effects of androgens on leptin and adiponectin production [148,152]. More importantly, they may be also directly and causally related to the differences in whole body insulin sensitivity and metabolism seen between sexes.

#### Sex steroids influence depot-specific adipose tissue metabolism and endocrine function

It is evident from the changes taking place during puberty and menopause, in women with polycystic ovary syndrome and in transgendered individuals that sex hormones have multiple effects of adipose tissue. The exact mechanisms involved remain largely obscure. Human adipocytes, as well as preadipocytes, express sex steroid receptors [154,155]. Both estrogens and androgens blunt lipolytic responses to catecholamines, an effect that is modulated at least partly via changes in adrenergic receptors expression [156,157], and also suppress LPL expression and activity [115,158]. Androgens have also been shown to increase glucose uptake [159]. Sex steroids have contradictory effects on leptin secretion - estrogens induce it and androgens inhibit it – in a sex specific manner, i.e. more so in adipocytes derived from women than from men [160,161]. It is also possible that sex steroids affect adipose tissue biology primarily via effects on the central nervous system, rather than via direct effects on the adipocytes. For example, in animal models, estrogen effects on steroidogenic factor-1 neurons of the ventromedial hypothalamic nucleus enhance brown adipose tissue thermogenesis and specifically limit visceral adipose tissue accumulation [162].

#### Sex steroids may regulate adipose tissue growth in a depot specific manner

Adipose tissue expands through enlargement of existing adipocytes and recruitment of progenitors. Sex differences in fat distribution involve both cell size and number: gluteo-femoral adipocytes of women are larger than in men, whereas abdominal adipocytes are comparable between sexes, and visceral adipocytes of women are smaller [105]. Nevertheless, the expansion of subcutaneous depots in obese women compared to men is mainly due to higher cell numbers [90,105]. More recently and with more exact imaging techniques, it was again shown that femoral fat accumulation in women is associated with increased adipocyte number (hyperplasia) whereas in men with increased adipocyte size (hypertrophy). Fat accumulation in the abdominal area is associated with hypertrophy in both sexes, but women start with more adipocytes even at the lean state and can therefore accommodate larger fat mass [163].

Although extensive studies document depot differences in the capacity of different depots of rodents to expand through hyperplasia [164,165], remarkably little is known about humans. Limited data suggest that the SAT of women, and particularly the femoral depot, contains a higher percentage of early differentiated adipocytes compared to men. Interestingly, *in vitro* proliferation and differentiation of preadipocytes isolated from the same individuals were comparable between sexes, leading these authors to suggest that the local microenvironment rather than inherent cellular differences promotes adipogenesis in women [166].

*In vitro*, estrogens stimulate proliferation of human preadipocytes [167,168], whereas androgens inhibit differentiation without affecting proliferation [158,169]. However, whether specific depots are differentially sensitive to sex steroid effects and potential sex-differences in response to these hormones are yet to be systemically investigated.

#### The systems biology of sex differences in adipose tissue

A comprehensive analysis of the gene networks that differ in ‘visceral’ adipose tissues (unspecified) of male and female mice clearly shows that gene networks identified in each sex affect different pathways and have different associations with metabolic and obesity traits (4). Recent studies in humans indicate the existence of sex biased mRNA and miRNA expression in abdominal and gluteal adipose tissues that will undoubtedly yield new mechanistic insights [170,171].

## Conclusions

Sex differences in the fat phenotypes are probably determined by a complex interplay of genetic, epigenetic, and hormonal factors. Elegant *in vivo* studies of depot- and sex-specific differences in adipose tissue metabolism showed that the primary suspects (lipid uptake and mobilization) are not the main mediators and at the same time pointed to new pathways (direct FFA uptake) for further investigation. We still do not know if sex differences in the function of female adipocytes are mainly derived from genetic, cell autonomous properties related to sex chromosomes or from critical early imprinting events by sex steroids. The direct effects of sex hormones on adipocyte function and the importance of the microenvironment of specific adipose depots on growth remain poorly understood. Much more work will be required to integrate all the data arising from studies of global gene and miRNA expression, as well as of epigenetic changes, and to understand why females can accumulate more adipose tissue than men without deleterious metabolic consequences, and how gluteal-femoral adipose tissue in particular lessens metabolic risk.

## Competing interests

The authors have no competing interests to disclose.

## Authors’ contributions

KK drafted and SKF edited the manuscript. SRS and ASG reviewed the manuscript and made suggestions. All authors read and approved the final manuscript.
